# The Dietary Polyphenol Resveratrol in Intestinal Ischemia–Reperfusion Injury: From Multifaceted Protective Mechanisms to Clinical Translation Challenges

**DOI:** 10.1002/fsn3.71666

**Published:** 2026-03-23

**Authors:** Xue‐feng Zhang, Zhi Huang, Yuan Zeng, Yan Zhang, Ping Li, Fei‐xiang Wang, Liang Nie

**Affiliations:** ^1^ Department of Anesthesiology Fushun County People's Hospital Zigong Sichuan China; ^2^ Department of General Surgery Dazhou Central Hospital Dazhou Sichuan China; ^3^ Department of Anesthesiology The Affiliated Hospital, Southwest Medical University Luzhou Sichuan China

**Keywords:** antioxidant, ferroptosis, inflammation, intestinal ischemia–reperfusion injury, resveratrol

## Abstract

Intestinal ischemia–reperfusion (I/R) injury constitutes a life‐threatening condition with mortality approaching 50%, yet effective therapeutic interventions remain limited. Resveratrol, a natural polyphenolic compound, has demonstrated promising multi‐targeted protective effects in preclinical models by simultaneously enhancing antioxidant defense, suppressing inflammatory cascades, inhibiting ferroptosis, stabilizing mast cells, and preserving intestinal barrier integrity through SIRT1/SIRT3‐mediated pathways. Novel delivery systems, including exosome‐based carriers and nanoformulations, have shown enhanced therapeutic efficacy in overcoming bioavailability limitations. However, a critical translational gap persists between experimental promise and clinical reality. Three interconnected obstacles impede progress: the complete absence of human clinical trials in intestinal I/R contexts, poor pharmacokinetics characterized by extensive first‐pass metabolism that raises fundamental questions about achievable therapeutic tissue concentrations, and the lack of large animal validation studies bridging rodent models and human pathophysiology. This review provides critical analysis of evidence quality, identifies specific knowledge gaps, and proposes a structured translational roadmap prioritizing clinically relevant post‐ischemic treatment paradigms, comprehensive pharmacokinetic characterization, biomarker development, and proof‐of‐concept clinical trials to advance resveratrol toward clinical benefit.

## Graphical abstract

Intestinal ischemia–reperfusion (I/R) injury is a life‐threatening condition with mortality approaching 50%, driven by oxidative stress, inflammatory cascades, ferroptosis, and intestinal barrier disruption that ultimately precipitates distant organ dysfunction. Resveratrol, a naturally occurring dietary polyphenol, exerts multifaceted protective effects against intestinal I/R injury through five interconnected mechanisms: (1) enhancing endogenous antioxidant defense by upregulating superoxide dismutase and glutathione peroxidase 4 to neutralize reactive oxygen species; (2) suppressing inflammatory signaling via inhibition of nuclear factor‐kappa B nuclear translocation and reduction of pro‐inflammatory cytokines; (3) activating SIRT1 and SIRT3 signaling networks that coordinate cellular stress responses and mitochondrial homeostasis; (4) inhibiting iron‐dependent ferroptotic cell death through the glutathione/glutathione peroxidase 4 axis; and (5) stabilizing mast cells to prevent degranulation‐mediated remote organ injury, particularly acute lung injury. Furthermore, resveratrol preserves intestinal epithelial barrier integrity by maintaining tight junction protein expression, including zonula occludens‐1, occludin, and claudin‐1, thereby attenuating bacterial translocation and systemic inflammatory propagation. Novel delivery systems, including exosome‐based carriers and optimized nanoformulations, further augment its therapeutic efficacy. Despite compelling preclinical evidence, critical translational barriers—encompassing the absence of human clinical trials, poor oral bioavailability due to extensive first‐pass metabolism, and the lack of large animal validation studies—remain to be systematically addressed before clinical application can be realized.

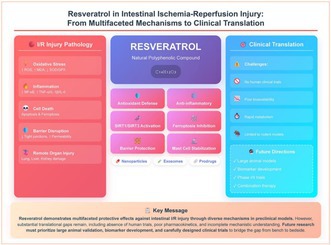

## 1 Introduction

Intestinal ischemia–reperfusion (I/R) injury constitutes a life‐threatening clinical condition characterized by substantial morbidity and mortality. This pathological entity occurs across diverse clinical contexts, encompassing acute mesenteric ischemia, intestinal obstruction, intussusception, small bowel transplantation, aortic aneurysm surgery, and cardiopulmonary bypass procedures (Lv et al. 2024; Pierro and Eaton 2004; Wang et al. 2024). Population‐based studies indicate that acute mesenteric ischemia, the most severe manifestation, occurs with an annual incidence of approximately 6.2–8.7 per 100,000 population, predominantly affecting elderly patients with multiple cardiovascular comorbidities (Kase et al. 2023; Tamme et al. 2022). The mortality associated with acute mesenteric ischemia approaches 50%, with particularly severe outcomes observed when intestinal I/R injury progresses to shock and multiple organ dysfunction syndrome (Lv et al. 2024; Pierro and Eaton 2004; Wang et al. 2024). Despite advances in surgical techniques and intensive care protocols, effective therapeutic strategies for intestinal I/R injury remain elusive, underscoring the critical necessity for novel protective agents (Wang et al. 2021). In this context, natural compounds with pleiotropic protective properties have emerged as promising therapeutic candidates.

Resveratrol, a naturally occurring polyphenolic compound predominantly derived from grape skins, red wine, and diverse plant species, has garnered considerable scientific interest owing to its pleiotropic pharmacological properties (Farhan and Rizvi 2023). This stilbenoid compound exhibits multifaceted biological activities, encompassing potent antioxidant, anti‐inflammatory, cardioprotective, and neuroprotective effects (Farhan and Rizvi 2023; Katila et al. 2022; Mohammadi et al. 2024). Accumulating preclinical evidence demonstrates that resveratrol ameliorates intestinal I/R injury through diverse mechanisms, including free radical scavenging, inflammatory response modulation, sirtuin (SIRT) pathway activation, and ferroptosis inhibition (Dong et al. 2013; Parlar and Arslan 2019; Wang et al. 2023). Furthermore, resveratrol has been shown to restore intestinal barrier integrity, attenuate bacterial translocation, and confer protection against remote organ damage subsequent to intestinal I/R (Ozkan et al. 2009; Yildiz et al. 2009). Given this compelling preclinical evidence, a comprehensive synthesis of current knowledge is warranted to guide future translational efforts.

Therefore, the present review systematically examines the current understanding of resveratrol's protective mechanisms in intestinal I/R injury, critically evaluating experimental evidence pertaining to its antioxidant properties, modulation of cellular signaling networks, regulation of cell death pathways, and preservation of intestinal barrier function. Additionally, we critically appraise the quality of existing evidence, discuss challenges inherent to clinical translation, and delineate pivotal knowledge gaps warranting future investigation.

## 2 Literature Search and Selection Strategy

This scoping review was conducted following the Preferred Reporting Items for Systematic reviews and Meta‐Analyses extension for Scoping Reviews (PRISMA‐ScR) guidelines. A comprehensive literature search was performed in PubMed, Web of Science, and Scopus databases from inception to December 2025. The search strategy combined Medical Subject Headings (MeSH) terms and keywords: (“resveratrol” OR “trans‐resveratrol” OR “polyphenol”) AND (“intestinal ischemia–reperfusion” OR “intestinal I/R” OR “mesenteric ischemia” OR “intestinal reperfusion injury”) AND (“mechanism” OR “protective effect” OR “antioxidant” OR “inflammation” OR “ferroptosis” OR “SIRT” OR “barrier function”).

Studies were included if they: (1) investigated resveratrol's effects in intestinal I/R injury models; (2) reported mechanistic data or therapeutic outcomes; (3) were published in English; and (4) were original research articles or clinical trials. Exclusion criteria comprised: (1) studies focusing exclusively on other organ I/R injuries without intestinal involvement; (2) reviews, editorials, or conference abstracts; and (3) studies examining polyphenolic compounds other than resveratrol as the primary intervention. Two reviewers independently screened titles and abstracts, followed by full‐text assessment of potentially eligible articles. Disagreements were resolved through discussion. Reference lists of included studies were hand‐searched to identify additional relevant publications. Data extraction focused on study design, animal models, resveratrol dosing regimens, protective mechanisms, outcome measures, and translational implications. Given the heterogeneity in experimental protocols and outcome measures, a narrative synthesis approach was employed rather than meta‐analysis.

## 3 Pathophysiology of Intestinal I/R

The pathophysiological cascade of intestinal I/R injury encompasses intricate cellular and molecular perturbations orchestrated through multiple interconnected mechanisms. Central to this pathological process is oxidative stress, which emerges as a pivotal mediator of tissue damage. Specifically, during the ischemic phase, interrupted blood supply precipitates cellular energy depletion, adenosine triphosphate breakdown, and accumulation of hypoxanthine alongside conversion of xanthine dehydrogenase to xanthine oxidase (Li et al. 2022). Upon reperfusion, the readmission of oxygen triggers explosive reactive oxygen species (ROS) generation through multiple enzymatic sources including xanthine oxidase, nicotinamide adenine dinucleotide phosphate (NADPH) oxidase, mitochondrial electron transport chain, and uncoupled nitric oxide synthase (Li et al. 2022; Wu et al. 2018). These ROS inflict extensive damage to cellular macromolecules, including lipid membranes, proteins, and DNA, while simultaneously disrupting the delicate balance of endogenous antioxidant systems such as superoxide dismutase (SOD), catalase, and glutathione (GSH) peroxidase (GPX) (Hu et al. 2018; Li et al. 2022; Wu et al. 2018). Lipid peroxidation products, particularly malondialdehyde (MDA) and 4‐hydroxynonenal, serve as biomarkers of oxidative injury and contribute to propagation of cellular damage (Archontakis‐Barakakis et al. 2025).

In parallel with oxidative damage, oxidative stress initiates and amplifies a robust inflammatory cascade characterized by activation of critical transcriptional pathways and release of pro‐inflammatory mediators. The nuclear factor‐kappa B (NF‐κB) signaling pathway, activated through Toll‐like receptor recognition of damage‐associated molecular patterns and bacterial products, drives transcriptional upregulation of pro‐inflammatory cytokines including tumor necrosis factor‐alpha (TNF‐α), interleukin‐1 beta (IL‐1β), and interleukin‐6 (IL‐6) (Archontakis‐Barakakis et al. 2025; Jin et al. 2022). Simultaneously, enhanced expression of endothelial adhesion molecules, particularly intercellular adhesion molecule‐1 (ICAM‐1), facilitates neutrophil recruitment and sequestration within injured intestinal tissue (Olanders et al. 2002). These infiltrating neutrophils, activated by mesenteric lymph‐borne inflammatory factors, generate additional ROS and release cytotoxic proteases, establishing a deleterious feedback loop that perpetuates tissue injury (Archontakis‐Barakakis et al. 2025; Jin et al. 2022; Olanders et al. 2002). Elevated myeloperoxidase (MPO) activity in damaged mucosa reflects the magnitude of neutrophil infiltration and correlates with injury severity (Wang et al. 2024).

Beyond oxidative stress and inflammation, intestinal I/R injury triggers diverse cell death mechanisms that collectively contribute to mucosal devastation. Beyond classical apoptosis, which manifests during later reperfusion phases through activation of caspase cascades and mitochondrial permeabilization (Ikeda et al. 1998), emerging evidence highlights ferroptosis as a critical early contributor to intestinal epithelial cell death (Wang et al. 2024). Ferroptosis, an iron‐dependent regulated cell death pathway characterized by lipid peroxidation accumulation, occurs prominently during early reperfusion when ischemia‐induced alterations in cellular metabolism converge with restored oxygen availability (Wang et al. 2024). Depletion of GPX4 and reduced GSH levels compromise cellular antioxidant defenses, rendering cells vulnerable to ferroptotic death (Guo et al. 2023; Wang et al. 2024). These distinct cell death modalities exhibit temporal segregation and mechanistic interdependence, collectively contributing to progressive mucosal injury.

Moreover, disruption of intestinal barrier integrity constitutes a hallmark consequence of I/R injury with profound systemic implications. Ischemia‐induced hypoxia and subsequent reperfusion‐mediated oxidative stress compromise epithelial tight junction architecture through downregulation and redistribution of key structural proteins including zonula occludens‐1 (ZO‐1), occludin, and claudin family members (Li et al. 2025). This architectural disassembly increases paracellular permeability, facilitating translocation of bacteria, endotoxins, and pro‐inflammatory mediators from the intestinal lumen into systemic circulation (Lv et al. 2024; Wang et al. 2024). Additionally, mucus barrier impairment due to goblet cell dysfunction and reduced Muc2 secretion eliminates a critical protective layer, exposing the epithelium to luminal threats (Szandruk‐Bender et al. 2025). Elevated serum levels of intestinal fatty acid‐binding protein, diamine oxidase, and D‐lactate serve as biomarkers of epithelial damage and barrier dysfunction (Deng et al. 2023).

**FIGURE 1:**
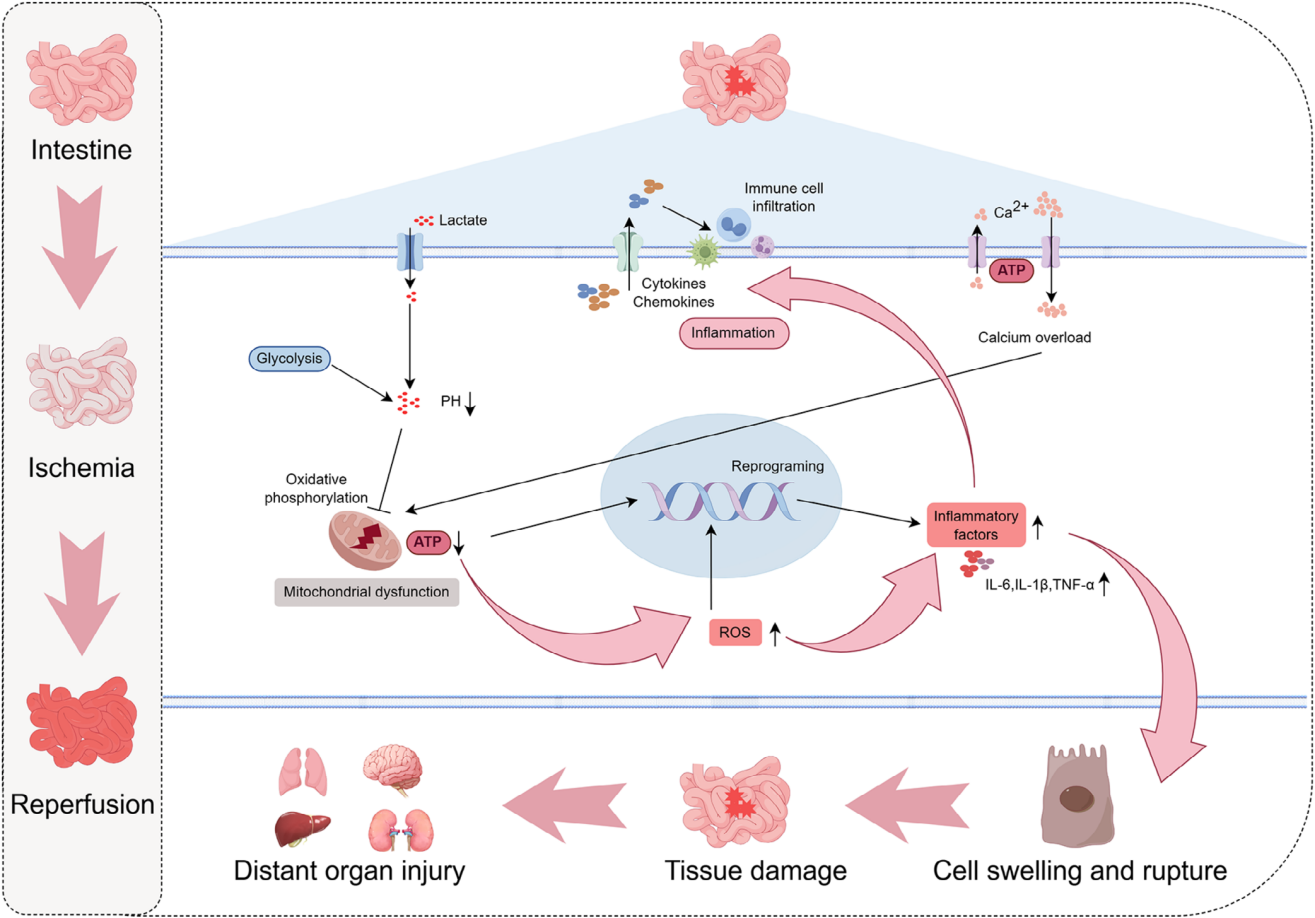
Pathophysiological cascade of intestinal I/R leading to distant organ damage. The figure illustrates the biphasic process of intestinal I/R injury and its systemic consequences. During the ischemic phase, impaired oxygen delivery triggers glycolysis with subsequent lactate accumulation and pH reduction. Cellular energy depletion leads to mitochondrial dysfunction, characterized by decreased ATP production and oxidative phosphorylation impairment. The reperfusion phase paradoxically amplifies tissue injury through calcium overload and excessive ROS generation. These metabolic derangements converge on cellular reprogramming, inducing production of inflammatory factors including IL‐6, IL‐1β, and TNF‐α. The amplified inflammatory response manifests as immune cell infiltration into intestinal tissue, mediated by cytokines and chemokines. Ultimately, this cascade progresses through cell swelling and rupture to localized tissue damage, culminating in distant organ injury affecting multiple organs including the lungs, liver, brain, and kidneys. ATP, adenosine triphosphate; Ca^2+^, calcium ion; IL‐1β, interleukin‐1 beta; IL‐6, interleukin‐6; I/R, ischemia–reperfusion; PH, potential of hydrogen; ROS, reactive oxygen species; TNF‐α, tumor necrosis factor‐alpha.

Importantly, the consequences of intestinal I/R extend beyond local injury to inflict damage upon distant organs, particularly the lungs, liver, and kidneys, culminating in systemic inflammatory response syndrome and multiple organ dysfunction syndrome (Fan et al. 2024). Mesenteric lymphatic drainage serves as a critical conduit transporting intestinal‐derived inflammatory mediators, damage‐associated molecular patterns, and activated immune cells into systemic circulation (Dzieciatkowska et al. 2014). In the pulmonary vasculature, these factors activate endothelial cells and resident immune populations, promoting neutrophil infiltration, microvascular hyperpermeability, and inflammatory cytokine production, thereby manifesting as acute lung injury (Lv et al. 2024). Similarly, hepatic injury emerges through neutrophil extracellular trap formation, enhanced ICAM‐1 expression, and cytokine‐mediated hepatocyte damage (Hayase et al. 2019). This systemic inflammatory propagation underscores the gut's role as a potential “motor” of multiple organ failure, with intestinal barrier dysfunction serving as the proximal trigger for downstream catastrophic events (Lv et al. 2024; Pierro and Eaton 2004; Wang et al. 2024, 2021). A concise summary of the pathogenesis of intestinal I/R and its consequent multi‐organ dysfunction is depicted in Figure 1.

Given the complexity and severity of these pathophysiological mechanisms, therapeutic strategies capable of targeting multiple injury pathways simultaneously are urgently needed. Among various candidate compounds, resveratrol has emerged as a promising multi‐targeted protective agent, as discussed in the following section.

## 4 Protective Mechanisms and Therapeutic Applications of Resveratrol in Intestinal I/R

### 4.1 Antioxidant Defense and Inflammatory Response Modulation

Resveratrol exerts profound protective effects against intestinal I/R injury predominantly through its potent antioxidant capacity and anti‐inflammatory properties. Multiple experimental studies have consistently demonstrated that resveratrol administration significantly attenuates oxidative stress by directly scavenging free radicals and enhancing endogenous antioxidant systems (Ozkan et al. 2009; Yildiz et al. 2009). Specifically, resveratrol treatment markedly reduces tissue levels of MDA, a prominent lipid peroxidation marker, and nitric oxide (NO), while simultaneously restoring activities of SOD, catalase, and GPX to physiological levels (Ozkan et al. 2009; Parlar and Arslan 2019; Yildiz et al. 2009). The compound effectively ameliorates intestinal mucosal injury, as evidenced by improved histopathological scores and reduced systemic release of injury markers including lactate dehydrogenase and aminotransferases (Borges et al. 2018; Parlar and Arslan 2019; Yildiz et al. 2009). Furthermore, resveratrol substantially decreases MPO activity, reflecting diminished neutrophil infiltration and inflammatory cell accumulation within damaged intestinal tissue (Borges et al. 2018; Ozkan et al. 2009; Parlar and Arslan 2019; Yildiz et al. 2009).

Beyond its antioxidant properties, the anti‐inflammatory mechanisms of resveratrol extend to encompass comprehensive modulation of inflammatory cascades. Resveratrol treatment significantly suppresses the production and secretion of pro‐inflammatory cytokines including TNF‐α and IL‐1β, thereby attenuating the systemic inflammatory response triggered by intestinal I/R injury (Parlar and Arslan 2019). Notably, resveratrol preserves intestinal smooth muscle contractile function, which typically deteriorates following I/R, by normalizing potassium chloride‐ and acetylcholine‐induced contractile responses at the late (24 h) reperfusion time point (Parlar and Arslan 2019). This functional recovery correlates with reduced oxidative stress and inflammatory mediator levels, underscoring the interconnected nature of resveratrol's protective mechanisms and its capacity to restore tissue homeostasis following ischemic insult.

### 4.2 SIRT Pathway Activation and Ferroptosis Inhibition

While antioxidant and anti‐inflammatory effects represent fundamental protective mechanisms, emerging evidence has identified SIRT signaling pathways as critical mediators of resveratrol's protective effects in intestinal I/R injury, with particular emphasis on SIRT1 and SIRT3 isoforms. Resveratrol functions as an agonist of SIRTs, NAD^+^‐dependent deacetylases that regulate cellular metabolism, oxidative stress responses, and inflammatory signaling (Dong et al. 2013; Wang et al. 2023). In subacute intestinal I/R models, resveratrol treatment significantly upregulates SIRT1 expression while concurrently inhibiting NF‐κB translocation and activity (Dong et al. 2013). This SIRT1‐mediated suppression of NF‐κB attenuates the transcriptional activation of inducible nitric oxide synthase (iNOS), consequently reducing pathological NO production (Dong et al. 2013). Pharmacological studies employing SIRT1 inhibitor nicotinamide confirm that resveratrol's protective efficacy is substantially diminished upon SIRT1 blockade, establishing the SIRT1‐NF‐κB‐iNOS‐NO axis as an essential mechanism underlying resveratrol's therapeutic actions (Dong et al. 2013).

Furthermore, genetic deletion studies in Sirt3^−/−^ mice demonstrate exacerbated intestinal mucosal ferroptosis, more severe I/R injury, and complete abrogation of resveratrol's protective effects, definitively establishing SIRT3 as an indispensable mediator (Wang et al. 2023). Importantly, the SIRT pathway's influence extends beyond intracellular metabolic regulation to encompass preservation of epithelial barrier architecture. Resveratrol maintains expression of tight junction proteins including ZO‐1 and occludin through SIRT‐dependent mechanisms, thereby preventing paracellular permeability and bacterial translocation (Wang et al. 2023). This barrier‐protective effect represents a critical link between intracellular signaling and tissue‐level integrity, warranting detailed examination of resveratrol's comprehensive effects on intestinal barrier function and immunomodulation.

### 4.3 Intestinal Barrier Protection and Immunomodulatory Effects

In addition to modulating intracellular signaling pathways, preservation of intestinal barrier integrity constitutes a critical therapeutic target in I/R injury management, and resveratrol demonstrates multifaceted barrier‐protective properties. Beyond its direct effects on tight junction proteins, resveratrol significantly attenuates bacterial translocation to mesenteric lymph nodes, liver, and spleen, thereby preventing gut‐derived sepsis and systemic inflammatory complications (Ozkan et al. 2009). This barrier‐protective effect is mechanistically linked to resveratrol's capacity to modulate immune cell function and polarization. Recent investigations employing resveratrol‐primed adipose‐derived stem cell exosomes (RSV‐primed Exo) reveal enhanced therapeutic efficacy compared to unmodified exosomes (Ye et al. 2025). RSV‐primed Exo treatment effectively mitigates pathological damage characterized by mucosal villous edema and inflammatory cell infiltration, while substantially restoring expression of tight junction proteins including ZO‐1, occludin, and claudin‐1 (Ye et al. 2025).

Mechanistically, the immunomodulatory effects underlying these barrier‐protective properties involve regulation of macrophage polarization from pro‐inflammatory M1 phenotype toward anti‐inflammatory M2 phenotype, mediated through inhibition of the NF‐κB signaling pathway (Ye et al. 2025). This macrophage phenotype shift results in decreased production of pro‐inflammatory cytokines (IL‐1β, IL‐6, TNF‐α) and increased secretion of anti‐inflammatory mediator IL‐10, creating an immunological microenvironment conducive to tissue repair (Ye et al. 2025). Beyond local effects, resveratrol's immunomodulatory actions extend to influence systemic immune responses through the gut‐brain axis (Dou et al. 2019). In cerebral ischemia models, resveratrol modulates intestinal flora composition, promotes Th1/Th2 balance toward Th2 polarization, and skews Treg/Th17 balance toward regulatory T cells in small intestinal lamina propria (Dou et al. 2019). These intestinal immunological changes translate to reduced systemic pro‐inflammatory cytokine levels, decreased blood–brain barrier disruption, and diminished neurological deficits, exemplifying the far‐reaching consequences of resveratrol's gut‐directed immunomodulation (Dou et al. 2019).

### 4.4 Novel Delivery Systems and Special Clinical Applications

To enhance clinical translation potential, the therapeutic efficacy of resveratrol has been further augmented through development of advanced drug delivery systems and exploration of specific clinical scenarios. Nanoparticle‐based formulations have been investigated to overcome resveratrol's inherent limitations in bioavailability and intestinal epithelial retention (Borges et al. 2018). Resveratrol‐loaded poly(anhydride) nanoparticles demonstrate comparable protective efficacy to free resveratrol in preventing oxidative stress, preserving myenteric neurons, and maintaining gastrointestinal transit in I/R models (Borges et al. 2018). However, critical safety concerns have emerged, as empty poly(anhydride) nanoparticles exhibit hepatotoxicity potentially attributed to nanoparticle translocation across compromised intestinal barriers, necessitating careful selection of carrier materials for clinical translation (Borges et al. 2018). Alternative polymeric delivery systems, such as resveratrol‐loaded poly(ethylene glycol)‐poly(phenylalanine) (RES/PEG‐PPhe) complexes, have shown promising results particularly in diabetic I/R models (Wang et al. 2019). This formulation demonstrates superior efficacy compared to free resveratrol in reducing pathological damage, decreasing intestinal edema, and improving metabolic parameters including blood glucose control (Wang et al. 2019).

Beyond delivery system optimization, specialized clinical applications reveal resveratrol's adaptability across diverse pathophysiological contexts. In diabetic rats, RES/PEG‐PPhe complexes uniquely modulate the interaction between cystathionine‐γ‐lyase (CSE)/hydrogen sulfide (H_2_S) and iNOS/NO pathways, enhancing tissue H2S levels while suppressing pathological NO production (Wang et al. 2019). This dual pathway modulation, combined with enhanced antioxidant capacity (increased SOD and GSH) and reduced oxidative stress markers (decreased MDA), provides comprehensive protection tailored to diabetic metabolic derangements (Wang et al. 2019). Intravenous administration strategies have also been explored, with low‐dose continuous infusions (0.056–0.28 mg/kg) demonstrating significant protective effects in severe I/R models involving 90‐min ischemia and 120‐min reperfusion (Petrat and de Groot 2011). Notably, while even the lowest tested dose (0.056 mg/kg) effectively reduced macroscopic damage scores, tissue MPO activity, and histopathological injury severity, resveratrol administration induces hypotensive effects requiring supplemental isotonic saline, highlighting the necessity for careful hemodynamic monitoring during clinical application (Petrat and de Groot 2011). Moreover, investigations of mucosal regeneration potential reveal that while resveratrol fails to enhance Ki‐67‐positive cell proliferation following I/R, alternative agents such as sodium pyruvate demonstrate superior capacity to facilitate epithelial proliferation and mucosal recovery (Brencher et al. 2017).

### 4.5 Mast Cell Stabilization and Remote Organ Protection

Among the diverse protective mechanisms identified, a particularly intriguing and clinically relevant mechanism involves resveratrol‐mediated stabilization of mast cells, which prevents degranulation and subsequent inflammatory cascade activation. Intestinal I/R injury triggers mast cell activation and degranulation, releasing preformed mediators including tryptase, β‐hexosaminidase, and histamine, which propagate both local and distant organ injury (Huang et al. 2017). Resveratrol pretreatment (15 mg/kg intraperitoneally for 5 days) effectively stabilizes mast cell membranes, preventing degranulation and reducing release of these inflammatory mediators (Huang et al. 2017). This mast cell‐stabilizing effect proves critical for protecting remote organs, particularly the lungs, from I/R‐induced acute lung injury (Huang et al. 2017). Resveratrol significantly improves pulmonary function, increases surfactant protein‐C expression, reduces pulmonary oxidative stress markers (MDA, MPO), and decreases expression of NADPH oxidase subunits as well as adhesion molecules (Huang et al. 2017).

**FIGURE 2:**
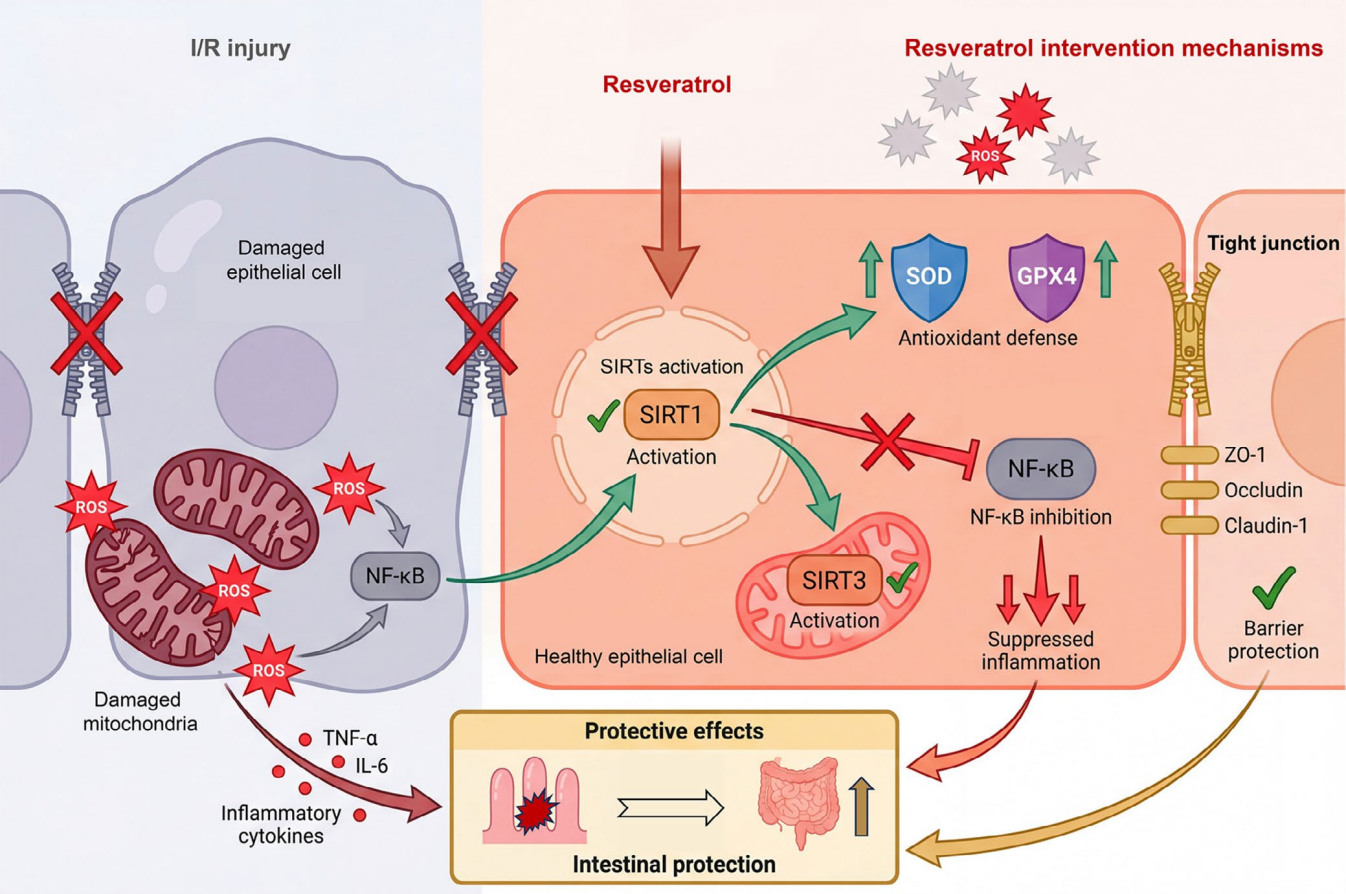
Multifaceted protective mechanisms of resveratrol in intestinal I/R. The figure illustrates how resveratrol intervenes in the pathological cascade from cellular damage to intestinal protection through four interconnected mechanisms. On the left, intestinal I/R injury initiates oxidative stress through mitochondrial dysfunction and excessive ROS generation, accompanied by NF‐κB activation and inflammatory cytokine production (TNF‐α and IL‐6). The disruption of tight junction proteins leads to barrier dysfunction. In the center, resveratrol enters intestinal epithelial cells and activates multiple protective pathways. First, resveratrol activates SIRT1 in the nucleus and SIRT3 in mitochondria, serving as upstream regulators of cellular stress responses. Second, resveratrol enhances antioxidant defense by upregulating SOD and GPX4 enzymes, effectively neutralizing ROS and preventing oxidative damage. Third, resveratrol inhibits NF‐κB nuclear translocation, thereby suppressing the inflammatory cascade and reducing pro‐inflammatory cytokine production. Fourth, resveratrol preserves intestinal barrier integrity by maintaining tight junction protein expression, including ZO‐1, occludin, and claudin‐1. The bottom panel demonstrates the final protective outcome: preserved intestinal architecture and reduced tissue damage. GPX4, glutathione peroxidase 4; I/R, ischemia–reperfusion; IL‐6, interleukin‐6; NF‐κB, nuclear factor kappa B; ROS, reactive oxygen species; SIRT1, sirtuin 1; SIRT3, sirtuin 3; SOD, superoxide dismutase; TNF‐α, tumor necrosis factor‐alpha; ZO‐1, zonula occludens‐1.

Rigorous mechanistic validation studies employing mast cell stabilizer cromolyn sodium demonstrate comparable protective effects to resveratrol, while mast cell degranulator compound 48/80 completely abrogates resveratrol's protective actions, definitively establishing mast cell stabilization as a prerequisite for resveratrol's efficacy (Huang et al. 2017; Zhao et al. 2018). Within intestinal tissue, mast cell stabilization by resveratrol inhibits activation of the NLR family pyrin domain containing 3 (NLRP3) inflammasome, a critical intracellular signaling complex that processes pro‐IL‐1β and pro‐IL‐18 into their mature, bioactive forms (Zhao et al. 2018). Prevention of mast cell degranulation disrupts the TNF‐α‐mediated activation of NLRP3 inflammasomes in intestinal mucosal epithelial cells, consequently reducing production and secretion of IL‐1β and IL‐18 (Zhao et al. 2018). This multi‐level inhibition of inflammatory signaling represents a unique mechanism distinct from classical antioxidant or anti‐inflammatory pathways, highlighting resveratrol's capacity to interrupt pathological cascades at multiple regulatory nodes and thereby provide comprehensive protection against both local intestinal injury and remote organ complications (Huang et al. 2017; Zhao et al. 2018). A comprehensive summary of preclinical studies examining resveratrol's protective effects in intestinal I/R injury is presented in Table 1 and Figure 2.

**TABLE 1:** Summary of preclinical studies evaluating resveratrol in intestinal I/R.

Animal model	Intervention details	Primary mechanisms	Key findings	References
Male C57BL/6 mice; Sirt3^−/−^ mice	RSV 0.6 mg, i.g., daily for 14 days before I/R	SIRT3/FoxO3a pathway; Ferroptosis inhibition	↑ SIRT3, FoxO3a activation; ↓ mitochondrial ROS; ↓ lipid peroxidation; ↑ SOD2, catalase; ↓ ferroptosis; Preserved ZO‐1, occludin; Effect lost in Sirt3^−/−^ mice	Wang et al. (2023)
Male BALB/c mice	RSV 50 mg/kg, i.g., daily for 10 days before I/R	SIRT1/NF‐κB/iNOS/NO axis	↑ SIRT1 expression; ↓ NF‐κB translocation; ↓ iNOS expression; ↓ NO production; Effect abolished by nicotinamide (SIRT1 inhibitor)	Dong et al. (2013)
Male Wistar rats	RSV 15 mg/kg, i.p., daily for 5 days before I/R	Antioxidant effects; Anti‐inflammatory; Smooth muscle function preservation	↓ MDA, MPO, IL‐1β and TNF‐α; ↑ GSH	Parlar and Arslan (2019)
Female Wistar rats	RSV 15 mg/kg, i.p., daily for 5 days before I/R	Antioxidant defense; Anti‐inflammatory	↓ MDA, NO and MPO; ↑ SOD; ↓ Bacterial translocation to MLN, liver, spleen	Ozkan et al. (2009)
Male Wistar rats	RSV 15 mg/kg, i.p., single dose before I/R and before reperfusion	Antioxidant defense	↓ TOS, OSI and MPO; Improved histopathological scores	Yildiz et al. (2009)
Male Wistar rats	RSV‐loaded poly(anhydride) nanoparticles 7 mg/kg, i.g., 5 days before surgery and continued for 7 days after surgery	Antioxidant defense; Myenteric neuron preservation; Drug delivery system	Preserved myenteric neurons; Maintained GI transit; Safety concern: Empty nanoparticles showed hepatotoxicity	Borges et al. (2018)
Male C57BL/6 mice	RSV‐primed ADSC‐derived exosomes, i.v., once after reperfusion	Macrophage polarization (M1 → M2); NF‐κB inhibition; Barrier protection	↓ mucosal villous edema; ↑ ZO‐1, occludin, claudin‐1; M2 macrophage polarization; ↓ IL‐1β, IL‐6, TNF‐α; ↑ IL‐10; Superior to unmodified exosomes	Ye et al. (2025)
Male C57BL/6 mice (cerebral ischemia model)	RSV 200 mg/kg, i.p., daily for 3 days after cerebral ischemia	Gut‐brain axis modulation; Intestinal flora regulation; Immune balance	Modulated intestinal flora; Th1/Th2 balance toward Th2; Treg/Th17 balance toward Treg in small intestinal lamina propria; ↓ systemic pro‐inflammatory cytokines; ↓ BBB disruption; ↓ neurological deficits	Dou et al. (2019)
Male SD diabetic rats	RSV‐loaded PEG‐PPhe complex 20 mg/kg, subcutaneous injection, daily for 3 days before I/R	CSE/H_2_S and iNOS/NO pathway interaction; Enhanced antioxidant capacity	↑ H_2_S levels; ↓ NO production; ↑ SOD, GSH; ↓ MDA; ↓ pathological damage; ↓ intestinal edema; Improved blood glucose control; Superior to free RSV in diabetic model	Wang et al. (2019)
Male Wistar rats	RSV 0.056–0.28 mg/kg, i.v., continuous infusion during I/R	Antioxidant	↓ macroscopic damage scores; ↓ MPO activity; ↓ histopathological injury; Effective even at minimal dose (0.056 mg/kg); Safety concern: Hypotensive effect requiring isotonic saline supplementation	Petrat and de Groot (2011)
Male Wistar rats	RSV (40 μg) compared with glycine and pyruvate, continuous infusion during I/R	Mucosal regeneration assessment	RSV failed to enhance Ki‐67‐positive cell proliferation; Sodium pyruvate showed superior mucosal regeneration capacity	Brencher et al. (2017)
Male Sprague–Dawley rats	RSV 15 mg/kg, i.p., daily for 5 days before I/R	Mast cell stabilization; Remote lung protection	↓ mast cell degranulation; ↓ tryptase; Improved pulmonary function; ↑ surfactant protein‐C; ↓ pulmonary MDA, MPO; ↓ NADPH oxidase subunits; Effect abolished by compound 48/80 (mast cell degranulator)	Huang et al. (2017)
Male Sprague–Dawley rats	RSV 15 mg/kg, i.p., daily for 5 days before I/R	Mast cell stabilization; NLRP3 inflammasome inhibition	↓ mast cell degranulation; ↓ TNF‐α; Inhibited NLRP3 inflammasome activation; ↓ IL‐1β, IL‐18 production; Comparable effect to cromolyn sodium (mast cell stabilizer)	Zhao et al. (2018)

Abbreviations: ↑, increased/upregulated; ↓, decreased/downregulated; ADSC, adipose‐derived stem cells; BBB, blood–brain barrier; CSE, cystathionine‐γ‐lyase; FoxO3a, forkhead box O3a; GI, gastrointestinal; GSH, glutathione; H_2_S, hydrogen sulfide; i.g., intragastric; iNOS, inducible nitric oxide synthase; i.p., intraperitoneal; i.v., intravenous; I/R, ischemia–reperfusion; MDA, malondialdehyde; MLN, mesenteric lymph nodes; MPO, myeloperoxidase; NF‐κB, nuclear factor kappa B; NLRP3, NLR family pyrin domain containing 3; NO, nitric oxide; OSI, oxidative stress index; PEG‐PPhe, poly(ethylene glycol)‐poly(phenylalanine); ROS, reactive oxygen species; RSV, resveratrol; SIRT, sirtuin; SOD, superoxide dismutase; TNF‐α, tumor necrosis factor alpha; TOS, total oxidative status; ZO‐1, zonula occludens‐1.

Collectively, these preclinical studies demonstrate that resveratrol exerts protective effects through multiple interconnected mechanisms spanning antioxidant defense, anti‐inflammatory actions, cell death regulation, barrier preservation, and immune modulation. However, critical evaluation of the quality and scope of this evidence base is essential to guide future translational efforts, as discussed below.

## 5 Critical Appraisal: Evidence Quality, Limitations, and Translational Barriers

### 5.1 Quality of Current Evidence and Methodological Considerations

The existing evidence base supporting resveratrol's protective effects in intestinal I/R injury derives predominantly from preclinical rodent studies employing well‐established superior mesenteric artery occlusion models (Gonzalez et al. 2015a). Multiple independent investigations demonstrate remarkable consistency in core findings, confirming resveratrol's capacity to attenuate oxidative stress, suppress inflammatory responses, and preserve intestinal barrier integrity (Dong et al. 2013; Ozkan et al. 2009; Parlar and Arslan 2019; Wang et al. 2023; Yildiz et al. 2009). However, substantial methodological heterogeneity compromises direct cross‐study comparisons and limits evidence synthesis. Ischemic durations range from 30 to 90 min, reperfusion periods vary from 30 min to 72 h, and resveratrol dosing regimens differ markedly in route of administration (oral, intraperitoneal, intravenous), dosage (0.056–200 mg/kg), treatment duration (single dose to 14 days of pretreatment), and timing relative to ischemic insult (Borges et al. 2018; Brencher et al. 2017; Dong et al. 2013; Dou et al. 2019; Huang et al. 2017; Ozkan et al. 2009; Parlar and Arslan 2019; Petrat and de Groot 2011; Wang et al. 2023, 2019; Ye et al. 2025; Yildiz et al. 2009; Zhao et al. 2018). Sample sizes typically range from 6 to 10 animals per group, potentially limiting statistical power for detecting subtle effects.

### 5.2 Fundamental Limitations and Critical Knowledge Gaps

Beyond methodological heterogeneity, several fundamental limitations constrain clinical translation. The most critical deficiency is the complete absence of human clinical trials evaluating resveratrol in intestinal I/R contexts (Gonzalez et al. 2015a). While resveratrol has undergone clinical testing for cardiovascular disease, neurological disorders, and metabolic syndrome, no studies have addressed acute intestinal ischemic events (Brown et al. 2024; Ko et al. 2017). This gap leaves fundamental questions unanswered regarding appropriate human dosing, optimal administration timing, safety profiles in critically ill patients, and actual clinical efficacy.

The temporal disconnect between experimental design and clinical reality represents a particularly critical limitation. The predominance of preventive treatment paradigms, wherein resveratrol is administered days to weeks prior to ischemic insult, sharply contrasts with clinical scenarios where therapeutic intervention necessarily occurs after ischemia onset (Gonzalez et al. 2015a). This experimental‐clinical mismatch likely overestimates therapeutic efficacy, as preventive dosing allows cellular preconditioning mechanisms that would be unavailable in acute treatment contexts. Studies employing post‐ischemic resveratrol administration remain scarce, and those that exist typically administer treatment immediately upon reperfusion rather than after diagnostic delays inherent to clinical practice.

The absence of large animal validation studies constitutes another critical gap. Porcine models, which more faithfully recapitulate human intestinal anatomy, vascular architecture, immune responses, and metabolic physiology, would provide essential bridging data between rodent experiments and human applications (Gonzalez et al. 2015b). Current evidence derives exclusively from small rodents, whose fundamental physiological differences from humans—including vastly different metabolic rates, immune system organization, and drug disposition—limit translational predictive value.

### 5.3 Pharmacokinetic Barriers and Bioavailability Challenges

Resveratrol exhibits notoriously poor oral bioavailability due to extensive first‐pass metabolism through glucuronidation and sulfation pathways, resulting in rapid conversion to metabolites with uncertain biological activity (Brown et al. 2024; Ko et al. 2017; Smoliga et al. 2011). Peak plasma concentrations remain low even with high oral doses, and the parent compound is rapidly eliminated from circulation (Brown et al. 2024; Smoliga et al. 2011). This pharmacokinetic profile raises critical questions about achievable tissue concentrations in human intestinal mucosa and whether experimentally effective doses can be safely and practically administered to patients.

Novel drug delivery systems—including nanoparticle formulations, exosome carriers, and prodrug derivatives—show promise in preclinical studies but themselves require extensive safety validation before clinical application (Borges et al. 2018; Summerlin et al. 2015; Ye et al. 2025). The observation of nanocarrier hepatotoxicity with certain polymer formulations underscores the necessity for comprehensive toxicological evaluation (Borges et al. 2018).

### 5.4 Incomplete Mechanistic Understanding and Pathway Interactions

While individual protective mechanisms have been characterized, the relative contributions of distinct pathways and their temporal dynamics remain poorly understood. The interplay between antioxidant effects, SIRT pathway activation, ferroptosis inhibition, anti‐inflammatory actions, and mast cell stabilization requires elucidation (Dong et al. 2013; Huang et al. 2017; Wang et al. 2023; Ye et al. 2025; Zhao et al. 2018). Do these mechanisms operate synergistically, sequentially, or redundantly? Which pathways are essential versus contributory? Understanding these relationships is critical for rational combination therapy design and identifying biomarkers predictive of treatment response.

The field lacks validated biomarkers for predicting treatment response or monitoring therapeutic efficacy in real‐time (Chen and Liu 2025; Zhang et al. 2023). Without such tools, conducting rational dose‐finding studies or personalizing treatment approaches becomes exceedingly difficult. Moreover, current animal models employ young, healthy subjects without comorbidities, whereas clinical intestinal I/R typically affects elderly patients with multiple comorbidities including diabetes, atherosclerosis, chronic inflammation, and polypharmacy (Wang et al. 2019). How these comorbid conditions influence resveratrol's efficacy, safety, and pharmacokinetics remains entirely unexplored. Age‐related alterations in antioxidant capacity, inflammatory status, and drug metabolism could substantially modify treatment responses, yet no studies have systematically examined these variables.

## 6 Future Research Directions

Future investigations must prioritize bridging the translational gap between preclinical success and clinical application. Development of large animal models, particularly porcine systems that more accurately reflect human intestinal anatomy, vascular architecture, and immune responses, represents an essential intermediate step (Alicehajic et al. 2024; Gonzalez et al. 2015b). These models would enable more rigorous evaluation of clinically relevant treatment paradigms, including post‐ischemic therapeutic administration rather than prophylactic pretreatment. Simultaneously, parallel efforts should focus on developing and validating novel biomarkers for intestinal I/R injury that could facilitate early diagnosis and treatment monitoring (Chen and Liu 2025; Zhang et al. 2023). Metabolomic and metagenomic approaches have identified promising candidates including specific gut microbiota signatures, circulating exosomal markers, and metabolite panels that warrant prospective validation (Alicehajic et al. 2024; Chen and Liu 2025; Zhang et al. 2023). Integration of multi‐omics technologies with machine learning algorithms may enable identification of predictive biosignatures for treatment response and risk stratification (Chen and Liu 2025).

At the mechanistic level, future investigations should address unresolved questions regarding pathway interactions and temporal dynamics. Comprehensive studies examining the crosstalk between oxidative stress, inflammatory cascades, ferroptosis, and intestinal microbiota dysbiosis would illuminate therapeutic targets for combination interventions (Chen et al. 2022; Deng et al. 2022). The emerging recognition of gut microbiota as critical modulators of I/R injury opens new therapeutic avenues (Chen et al. 2022; Deng et al. 2022; Huang et al. 2022). Future research should explore whether resveratrol's protective effects partly stem from microbiome modulation and whether probiotics or fecal microbiota transplantation could synergistically enhance efficacy. Additionally, epigenetic mechanisms, including histone modifications and non‐coding RNA regulation, represent another frontier requiring systematic investigation. Understanding how resveratrol influences intestinal epithelial regeneration and stem cell function could inform strategies for promoting mucosal recovery beyond acute injury mitigation (Borges et al. 2018; Brencher et al. 2017).

From a pharmaceutical development perspective, efforts must prioritize overcoming bioavailability limitations through rational formulation design. Advanced drug delivery systems—including targeted nanoparticles, prodrug derivatives with enhanced metabolic stability, and alternative administration routes—require rigorous preclinical evaluation followed by Phase I safety studies in healthy volunteers (Smoliga et al. 2011; Summerlin et al. 2015). Chemical modifications that preserve pharmacological activity while improving pharmacokinetic properties, such as methylated derivatives or acetylated forms, merit systematic comparison (Brown et al. 2024; Smoliga et al. 2011). Alternatively, co‐administration strategies combining resveratrol with metabolism inhibitors or absorption enhancers could provide practical solutions without requiring novel formulations. Importantly, all pharmaceutical development must incorporate comprehensive safety assessments, including long‐term toxicity studies and evaluation of potential drug–drug interactions in polypharmacy scenarios common among critically ill patients.

Ultimately, clinical translation should proceed through carefully designed proof‐of‐concept studies in well‐defined patient populations. Initial Phase I trials should establish safe dosing ranges and characterize pharmacokinetics in critically ill patients, whose altered physiology may substantially impact drug disposition. Subsequent Phase II trials could evaluate feasibility and preliminary efficacy signals in specific clinical contexts such as planned small bowel transplantation or high‐risk vascular surgery, where timing of intervention can be controlled. Subsequently, adaptive trial designs incorporating biomarker‐guided dose optimization would maximize information yield while minimizing patient exposure. Combination therapy approaches, pairing resveratrol with established critical care interventions, may prove more effective than monotherapy. Throughout this developmental pathway, emphasis must be placed on patient‐centered outcomes including organ function recovery, hospital length of stay, and quality of life, rather than solely surrogate biochemical markers. Only through such systematic, stepwise progression can resveratrol's promising preclinical profile be translated into meaningful clinical benefit for patients suffering intestinal I/R injury. In summary, while the path forward presents substantial challenges, the convergence of these research directions offers realistic potential for translating resveratrol's preclinical promise into clinical benefit.

## 7 Conclusions

The accumulated preclinical evidence convincingly demonstrates that resveratrol exerts multifaceted protective effects against intestinal I/R injury through diverse molecular mechanisms. These include potent antioxidant actions via enhancement of endogenous antioxidant systems, suppression of inflammatory cascades through NF‐κB pathway inhibition and macrophage polarization, activation of SIRT1/SIRT3 signaling networks, inhibition of ferroptotic cell death via the GSH/GPX4 axis, stabilization of mast cells to prevent remote organ injury, and preservation of intestinal barrier integrity through maintenance of tight junction proteins. Novel drug delivery systems, particularly exosome‐based carriers and optimized nanoformulations, have shown promise in enhancing therapeutic efficacy while addressing inherent bioavailability limitations. The breadth and consistency of these protective effects across multiple independent investigations establish resveratrol as a compelling candidate for further development as an intestinal I/R therapeutic agent.

Despite this compelling preclinical evidence, the journey from experimental potential to clinical actuality remains significant and must be navigated with scientific precision and realistic expectations. Critical knowledge gaps persist regarding optimal dosing regimens, treatment timing windows, long‐term safety profiles, and actual clinical efficacy in human patients. The absence of any human clinical trials in intestinal I/R contexts represents the most pressing limitation, one that cannot be addressed through additional animal studies alone. Furthermore, the complex pathophysiology of clinical intestinal I/R—occurring in elderly patients with multiple comorbidities, evolving over variable time courses, and presenting with diverse etiologies—differs markedly from controlled experimental models. Future research must prioritize translational studies employing large animal models, comprehensive pharmacokinetic characterization in relevant patient populations, development of validated biomarkers, and ultimately, carefully designed clinical trials. While resveratrol holds genuine promise as a potential therapeutic intervention for intestinal I/R injury, realizing this potential will require sustained, systematic investigation addressing the substantial challenges that separate preclinical success from clinical benefit.

## Author Contributions

X.Z., Z.H., F.W., and L.N.: conceptualization and methodology. X.Z., Z.H., Y.Z., Y.Z., P.L., F.W., and L.N.: data curation, formal analysis, and writing – original draft. All authors: read and approved the final manuscript.

## Funding

The authors have nothing to report.

## Ethics Statement

The authors have nothing to report.

## Conflicts of Interest

The authors declare no conflicts of interest.

## Data Availability

The authors have nothing to report.
